# Developing guidelines for school closure interventions to be used during a future influenza pandemic

**DOI:** 10.1186/1471-2334-10-221

**Published:** 2010-07-27

**Authors:** Nilimesh Halder, Joel K Kelso, George J Milne

**Affiliations:** 1 School of Computer Science and Software Engineering, University of Western Australia, Perth, Australia

## Abstract

**Background:**

The A/H1N1 2009 influenza pandemic revealed that operational issues of school closure interventions, such as *when *school closure should be initiated (activation trigger), *how long *schools should be closed (duration) and *what type *of school closure should be adopted, varied greatly between and within countries. Computer simulation can be used to examine school closure intervention strategies in order to inform public health authorities as they refine school closure guidelines in light of experience with the A/H1N1 2009 pandemic.

**Methods:**

An individual-based simulation model was used to investigate the effectiveness of school closure interventions for influenza pandemics with R_0 _of 1.5, 2.0 and 2.5. The effectiveness of *individual school closure *and *simultaneous school closure *were analyzed for 2, 4 and 8 weeks closure duration, with a daily diagnosed case based intervention activation trigger scheme. The effectiveness of combining antiviral drug treatment and household prophyaxis with school closure was also investigated.

**Results:**

Illness attack rate was reduced from 33% to 19% (14% reduction in overall attack rate) by 8 weeks school closure activating at 30 daily diagnosed cases in the community for an influenza pandemic with R_0 _= 1.5; when combined with antivirals a 19% (from 33% to 14%) reduction in attack rate was obtained. For R_0 _>= 2.0, school closure would be less effective. An 8 weeks school closure strategy gives 9% (from 50% to 41%) and 4% (from 59% to 55%) reduction in attack rate for R_0 _= 2.0 and 2.5 respectively; however, school closure plus antivirals would give a significant reduction (~15%) in over all attack rate. The results also suggest that an *individual school closure *strategy would be more effective than *simultaneous school closure*.

**Conclusions:**

Our results indicate that the particular school closure strategy to be adopted depends both on the disease *severity*, which will determine the *duration *of school closure deemed acceptable, and its *transmissibility*. For epidemics with a *low *transmissibility (R_0 _< 2.0) and/or *mild *severity, individual school closures should begin once a daily community case count is exceeded. For a *severe*, *highly transmissible *epidemic (R_0 _>= 2.0), long duration school closure should begin as soon as possible and be combined with other interventions.

## Background

There is a continuing threat of a future novel influenza pandemic having high morbidity (in terms of hospitalization) and mortality (in terms of case fatality) rates. Major pandemics occurred in the past century due to antigenic shift and reassortment of influenza viruses causing millions of deaths; among them 1918's caused the most death [1]. There were also influenza pandemics that were moderate in terms of mortality in 1957 and 1968 [2]. In 2009, influenza A/H1N1 virus, first identified in Mexico, rapidly circulated around the world causing an influenza pandemic [3]. The A/H1N1 2009 influenza pandemic has caused at least 16,455 deaths in 213 countries as of 28^th ^February, 2010 [4]. The 2009 influenza pandemic may cause public health authorities to review their pandemic mitigation guidelines in the light of the limited success in containing and controlling the pandemic. Therefore, improved pandemic guidelines are especially required for future *highly pathogenic *pandemics, such as may occur if a human transmissible H5N1 virus emerges.

The influenza pandemic guidelines of many countries [5-7] and the World Health Organization (WHO) [8] suggest a series of non-pharmaceutical and pharmaceutical interventions. Among those intervention strategies, school closure is commonly suggested as a key intervention strategy to slow down the spread of a pandemic within a community, particularly at the early stages of its advancement. The rationale for considering school closures as a frontline intervention is that children and young adults are thought to be the most susceptible to any influenza virus due to their high contact rates within school clusters and limited (or no) immunity to a circulating virus strain when compared to adults. The significance of school closure is also reflected in the A/H1N1 2009 influenza pandemic where about a 60% of cases infected with influenza A/H1N1 virus are 18 years old or younger [9], and many of the disease transmissions took place in school clusters [9-12]. In addition, school closure can be quickly adopted with a high degree of compliance. School closure strategies are therefore significant for controlling the spread of a pandemic within a community, by breaking the chain of disease transmission among school children and young adults. This frontline intervention is also intended to allow sufficient time for the distribution of antiviral drugs and development of new vaccines.

During the early progression phase of the A/H1N1 2009 influenza pandemic, school closure interventions and application of antiviral drugs for treatment and prophylaxis were implemented in Australia and other parts of the world [13-15]; however, in certain countries the activation of these measures appears to have been somewhat haphazard. Modelling studies [16,10,23] have shown that influenza virus strains which are more transmissible than A/H1N1 2009 influenza pandemic, would be susceptible to attack rate reduction through school closure strategies, and might be contained if combined with other social distancing and pharmaceutical interventions. Benefits, in terms of reduced attack rates (cumulative illness attack rate and daily incidence rate) and avoided hospitalizations and deaths, would be expected from closing schools during an influenza pandemic; however, school closure must be weighed against the potential high economic and social costs. Studies [9,24] suggested that a 12 weeks school closure might cost in range of 0.2% - 1.0% of GDP in the UK. Strong hesitance may be experienced in the implementing school closure unless the virus strain is severe, i.e. has a high case fatality ratio. In addition there is no clear agreement on operational issues such as, *when *school closure should be initiated (its activation trigger), *how long *schools should be closed (length of closure or duration) and *what *types of school closures (either *individual school closure *or *simultaneous school closure*) should be adopted. In this context, Cauchemez *et al. *reviewed historical approaches of school closure strategies as a public health policy in [9]. No modelling study has yet extensively and systematically evaluated the operational issues of school closure interventions in terms of how they impact on the effectiveness of alternative school closure strategies; the objective of this study is to do just this. In light of the varied school closure responses [11,14,15] to the A/H1N1 2009 influenza pandemic taken by many countries we believe that the outcomes from this detailed evaluation of school closure strategies will help better inform public health policy makers as to the optimal use of school closure measures during a future influenza pandemic.

## Methods

### Population model

A detailed simulation model of a real community of *Albany *(a small city in Western Australia) with ~ 30,000 population was used to simulate local epidemics for R_0 _values of 1.5, 2.0 and 2.5 with the dynamics of influenza A/H1N1 2009 swine flu virus. Details and an extensive methodological description of the population model used here was described in [21,23,25]. Using this model, we conducted stochastic, individual-based simulations of local epidemics, assuming that an average of one new infection per day was randomly introduced into the population for the whole epidemic. The simulation period was divided into 12 hour day/night cycles and during each simulation cycle a nominal location of each individual was calculated, taking into consideration the cycle type (day/night, weekday/weekend), the infection state of each individual and whether child supervision was needed to look after a child at home. Individuals residing in the same location during the same period of time (cycle) were assumed to come into potential infective contact.

### School Closure and Antiviral interventions

The school closure interventions modelled were *individual school closure *and *simultaneous school closure *strategies. In the *individual school closure *strategy, we assumed that upon a single diagnosed symptomatic case within a primary school, the whole school was closed; if there was one or two cases diagnosed in a high school only the class members of the affected class were isolated; and finally if there were more than two cases diagnosed in a high school the entire school was closed. However, this school closure policy was only activated when the daily number of diagnosed cases in the community reached an activation trigger. Cases occurring in schools before this time did not result in school closure. We also evaluated a *simultaneous school closure *strategy where all schools in the community were closed simultaneously, using a similar community activation trigger. School closure interventions were modelled for fixed durations from 2, 4 or 8 weeks. The intervention activation trigger was modelled in a way such that the intervention would come into effect when there was a certain reported number of diagnosed cases per day in the community (for example, after reported cases reached 20 per day the public health authority may announce 2 weeks of school closure).

We also evaluated the application of antiviral drugs to allow us to examine intervention strategies which combine school closure with antiviral treatment and prophylaxis, as occurred during the A/H1N1 2009 influenza pandemic [13]. We assume that 50% of symptomatic individuals were diagnosed and treated with antiviral drugs and their close contact household members were given antivirals for prophylaxis. We also assumed that the antivirals were distributed continually throughout the pandemic period, restricted to 1 course for treatment and maximum 2 courses for prophylaxis per individual. The antiviral administration strategy began once the reported cases in the community reached 10 per day. This would provide sufficient time to the public health authorities to become aware of the arrival of the epidemic in the community and allow time for antiviral distribution and so forth. Detailed modelling explanation and parameters for antiviral drug interventions are given in [26] with key details being repeated below.

### Transmission Model

The transmission probability that a susceptible individual would be infected by an infectious individual when the two came into contact was calculated according to the following transmission function, which takes into account the disease infectivity of the infectious individual ***I_i _***and the susceptibility of susceptible individual ***I_s _***at the time of contact. The transmission probability function is given as follows:

The baseline transmission coefficient ***β ***was initially chosen to give an epidemic with an attack rate of 17.4% which is consistent with seasonal influenza. To achieve simulations under a range of reproductive numbers, ***β ***was increase from this baseline value to achieve epidemics of various R_0 _magnitudes. Details of the procedure for estimating R_0 _calibrating ***β ***are given in [21].

The disease infectivity parameter **Inf(*I_i_*) **was set to 1 for symptomatic individuals at the peak period of infection and then to 0.5 for the rest of the infectivity period. The infectiousness of asymptomatic individuals was also assumed to be 0.5 and this applied to all infected individuals after the latent period but before onset of symptoms. The infection profile of a *symptomatic *individual was assumed to last for 6 days as follows: a 0.5 day latent period (with **Inf(*I_i_*) **set to 0) is followed by 1 day asymptomatic and infectious, where **Inf(*I_i_*) **is set to 0.5; then 2 days at peak infectiousness (with **Inf(*I_i_*) **set to 1.0); followed by 2.5 days reduced infectiousness (with **Inf(*I_i_*) **set to 0.5). For an infected but *asymptomatic *individual the whole infectious period (of 5.5 days) is at the reduced level of infectiousness with **Inf(*I_i_*) **set to 0.5. This infectivity profile is a simplification of the infectivity distribution found in a study of viral shedding [27]. Following infection an individual is assumed to be immune to re-infection for the duration of the simulation. We further assume that influenza symptoms develop one day into the infectious period [27], with 20% of infections being asymptomatic among children and 32% being asymptomatic among adults. These percentages were derived by summing the age-specific antibody titres determined in table five of [28]. Symptomatic individuals withdrew into the home with the following probabilities; adults 50% and children 90%, which is in keeping with the work of [16,17].

The susceptibility parameter **Susc(*I_s_*) **is a function directly dependent on the age of the susceptible individual. It captures age-varying susceptibility to transmission due to either partial prior immunity or age-related differences in contact behavior. To achieve a realistic age specific infection rate, the age-specific susceptibility parameters were calibrated against the serologic infection rates for seasonal H3N2 in 1977-1978 in Tecumseh, Michigan [29].

The antiviral efficacy factor AVF(*I_i_*, *I_s_*) = (1 - AVE*_i_*)*(1 - AVE*_s_*) represents the potential reduction in infectiousness of an infected individual (denoted by **AVE*_i_***) induced by antiviral treatment, and the reduction in susceptibility of a susceptible individual (denoted by **AVE*_s_***) induced by antiviral prophylaxis. When no antiviral intervention was administrated the values of both **AVE*_i _***and **AVE*_s _***were assumed to be 0, indicating no reduction in infectiousness or susceptibility. However, when antiviral treatment was being applied to the infectious individual the value of **AVE*_i _***was set at 0.66, capturing a reduction in infectiousness by factor of 66% [30]. Similarly, when the susceptible individual was undergoing antiviral prophylaxis the value of **AVE*_s _***was set to 0.85 indicating a reduction in susceptibility by a factor of 85% [30].

## Results

### Simulated characteristics of the epidemics under the un-mitigated scenarios

Three separate influenza epidemics with the basic reproductive number, R_0 _of 1.5, 2.0 and 2.5 were simulated using the Albany population model. The outcomes of the simulated epidemics varied stochastically due to the random location of infectious individuals seeded into the model as index cases and the probabilistic model of influenza transmission. We further assumed that a continuous influx of infectious individuals was introduced from outside of the simulation boundary at a rate of one infectious case per day to achieve a sustained epidemic for each simulation. Therefore we determined the results of all simulated epidemics from the average of 40 separate simulation runs, each with stochastic choices made using a different random number sequence. The mean cumulative illness attack rates (or attack rate) of 40 simulation runs were 33% (standard deviation 0.57%), 50% and 59% of the total simulated population corresponding to R_0 _values of 1.5, 2.0 and 2.5 respectively; while peak daily incidences were 120, 333 and 564 cases per 10,000 population. The mean serial intervals or generation times are 2.49 days, 2.36 days and 2.21 days corresponding to R_0 _values of 1.5, 2.0 and 2.5 respectively.

### Impact of duration on school closure interventions

**Figure 1:**
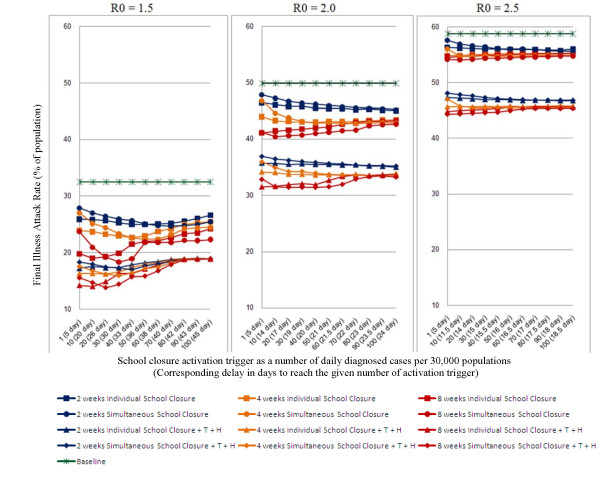
**Final illness attack rate of epidemics with school closure operational issues**. Outcomes of two different types of school closure intervention strategies (*individual school closure *and *simultaneous school closure*) for 2, 4 and 8 weeks school closure duration with/without antiviral treatment plus household prophylaxis (*T+H*) for three different R_0 _values of 1.5, 2.0 and 2.5 as a function of a number of daily diagnosed cases (activation trigger) are shown. The outcomes are reported in cumulative illness attack rate as a percentage of the simulated population size. The non-intervention or baseline epidemics are shown in green line. Three different colours have been used to report school closure periods; dark blue for 2 weeks, orange for 4 weeks and dark red for 8 weeks closure duration, using four different markers used to distinbuish the two types of school closure intervention and presence/absence of antiviral treatment plus household prophylaxis. We assumed that 50% of symptomatic cases would be diagnosed after 1 day of their symptom's appearance. We further assumed that antiviral treatment and prophylaxis (*T+H*) began after 10 cases were diagnosed in one day in the community.

The impact which different school closure durations have in reducing attack rate depends on the transmissibility of the particular virus strain, activation trigger and the type of school closure. The reduction in attack rate due to 2, 4 and 8 weeks school closure strategies for R_0 _of 1.5, 2.0 and 2.5 is shown in Figure 1.

For R_0 _= 1.5 a maximum of 8% (from 33% to 25%), 10% (from 33% to 23%) and 14% (from 33% to 19%) reduction in attack rate can be achieved at 2, 4 and 8 weeks of school closure respectively (see Figure 1 for R_0 _= 1.5; blue, orange and dark red lines with circle and square marker).

For R_0 _= 2.0, a maximum of a 5% (from 50% to 45%), a 7% (from 50% to 43%) and a 9% (from 50% to 41%) reduction in attack rate achieved for 2 weeks, 4 weeks and 8 weeks of school closure respectively (see Figure 1 for R_0 _= 2.0; blue, orange and dark red lines with circle and square marker).

Similarly for R_0 _= 2.5, a maximum of a 3% (from 59% to 56%), a 4% (from 59% to 55%) and 5% (from 59% to 54%) reduction in attack rate obtained for 2 weeks, 4 weeks and 8 weeks of school closure respectively (see Figure 1 for R_0 _= 2.5; blue, orange and dark red lines with circle and square marker).

Combining antiviral treatment and household prophylaxis (T+H) with school closure strategies decreases the attack rates for all duration scenarios. For example, for R_0 _= 2.0 and 2.5, ~10% reduction in attack rate would be achieved by coupling T+H with the school closure strategies compared to the purely school closure strategies (see Figure 1 for R_0 _= 2.0 and 2.5; comparing the results with T+H (diamond and triangle marker) and without T+H (circle and square marker) for all blue, orange and dark red lines); whereas for R_0 _= 1.5, ~8%, ~7% and ~5% reductions in attack rate would be achieved by 2, 4 and 8 weeks school closure strategies respectively (see Figure 1 for R_0 _= 1.5).

**Figure 2:**
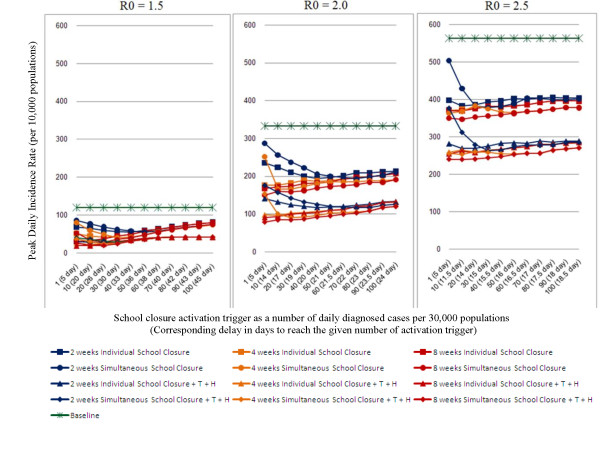
**Peak daily incidence rate of epidemics with school closure operational issues**. Outcomes of two different types of school closure intervention strategies (*individual school closure *and *simultaneous school closure*) for 2, 4 and 8 weeks school closure duration with/without antiviral treatment plus household prophylaxis (*T+H*) for three different R_0 _values of 1.5, 2.0 and 2.5 as a function of a number of daily diagnosed cases (activation trigger) are shown. The outcomes are reported in peak daily incidence rate per 10,000 of the population size, and assume a diagnosis ratio of 50%.

The effectiveness of school closure on the reduction in the *peak daily incidence *is shown in Figure 2 for the range of R_0_'s, activation triggers and durations considered. The pattern of reduction in the peak daily incidence is similar to that of the cumulative illness attack rate, although the reduction is larger in relative terms. The most notable difference is that the consequences of premature school closures are greater for the peak daily incidence, especially for R_0 _>= 2.0 (see Figure 2, blue lines with circle maker, centre and right panels).

Our simulation results show that for R_0 _= 1.5, a maximum reduction of 64 (from 120 to 56), 80 (from 120 to 40), 87 (from 120 to 33) cases per 10,000 population would be achieved by 2, 4 and 8 weeks school closure respectively. For R_0 _= 2.5, a maximum reduction of 184 (from 564 to 380), 194 (from 564 to 370), 214 (from 564 to 350) cases would be achieved by 2, 4 and 8 weeks school closure respectively (see Figure 2; dark blue, orange and dark red lines with circle and square marker).

Coupling antiviral treatment and household prophylaxis (T+H) with school closure strategies would further reduce the peak daily incidence. For example, at R_0 _= 2.5, a maximum reduction of 304 (from 564 to 260) cases per 10,000 would be achieved by 2 weeks school closure strategies with an activation trigger of 30 daily diagnosed cases (see Figure 2; dark blue lines with diamond and triangle marker).

### Impact of activation trigger on school closure interventions

The reductions in attack rate achievable by school closure reported above depend on schools closing at the right time. One of the observable measures of the advance of an influenza epidemic used in public heath forecasting is the number newly infected cases per day, as estimated by the reported number of daily diagnosed cases. In our simulation results, the reported number of daily diagnosed cases has been used as an activation trigger used to initiate the school closure interventions.

Our study indicates that the activation trigger which gives the maximum reduction in attack rate depends on R_0_, the closure duration, and the type of school closure intervention in a non-trivial way. The impact of the activation trigger on the reduction of attack rate is shown in Figure 1.

If schools close for 2 weeks an activation trigger of at least 50 cases per day gives an 8% (from 33% to 25%) reduction in attack for R_0 _= 1.5 (see Figure 1 for R_0 _= 1.5; blue lines with circle and square marker); whereas the optimal trigger is ~80 cases per day for the epidemics with R_0 _>= 2.0, (see Figure 1 for R_0 _>= 2.0; blue lines with circle and square marker).

For 4 weeks of school closure, activation triggers of 40, 50 and 10 cases per day should be used to obtain optimal attack rates (see Figure 1; orange lines with circle and square marker) for R_0 _of 1.5, 2.0 and 2.5 respectively. With 8 weeks of school closure, activation triggers of 30, 5 and 1 case(s) per day give the maximum reduction in attack rate for R_0_'s of 1.5, 2.0 and 2.5 respectively (see Figure 1; dark red lines with circle and square marker).

When adding antiviral treatment and household prophylaxis (T+H) to school closure strategies, simulation suggests that school closure activation triggers of 20, 40 and 50 cases per day are within 1% of optimal for R_0 _of 1.5, 2.0 and 2.5 respectively (see Figure 1; lines with diamond and triangle marker) and these activation triggers apply for all durations from 2 to 8 weeks. When antivirals are added to school closure, there is less variation in attack rate due to variation in the school closure activation trigger. This is because when antivirals are used, most (at least half, for the scenarios simulated) of the reduction in attack rate is due to antivirals, which have the same effect regardless of the school closure activation trigger. As a result, achieving optimal reduction in attack rates for the combined strategies is less dependant on precise choice of the activation trigger. It turns out that this is true to such a degree that for each R_0 _a single activation trigger can be chosen for all school closure durations that gives a final attack rate that is within 1% of the best possible outcome. The concurrent use of this antiviral strategy thus effectively eliminates the dependency of optimal activation trigger on school closure duration.

We have summarized the relationship between activation trigger (as the reported number of daily diagnosed cases), the proportion of population infected per day, the proportion of population infected within the community and the timing delay between the first infected individual appearing in the population, and the time at which interventions are initiated. The summarized results are given in Table 1.

**Table 1:** Relationship between activation trigger, cumulative diagnosed cases and intervention activation delay.

		R_0_					
		
Activation trigger		1.5		2.0		2.5	
		
Cases diagnosed per day	% of population diagnosed per day	Cumulative diagnosed cases (%)	Activation delay in days	Cumulative diagnosed cases (%)	Activation delay in days	Cumulative diagnosed cases (%)	Activation delay in days
1	0.003	1 (0.003)	5	1 (0.003)	5	1 (0.003)	5
5	0.017	19 (0.063)	14	14 (0.047)	11	12 (0.04)	9
10	0.033	53 (0.177)	20	40 (0.133)	14	31 (0.104)	11.5
15	0.05	97 (0.33)	24	60 (0.2)	16	48 (0.16)	13
20	0.067	136 (0.45)	26	83 (0.277)	17	68 (0.227)	14
25	0.083	190 (0.63)	28	109 (0.364)	18	85 (0.284)	14.5
30	0.1	237 (0.79)	30	136 (0.454)	19	99 (0.33)	15
40	0.13	335 (1.12)	33	188 (0.627)	20	140 (0.467)	15.5
50	0.167	454 (1.52)	36	235 (0.784)	21	179 (0.597)	16
60	0.2	586 (2.0)	38	288 (0.96)	21.5	214 (0.714)	16.5
70	0.233	708 (2.4)	40	345 (1.15)	22	245 (0.817)	17
80	0.267	848 (2.83)	42	409 (1.364)	23	298 (0.994)	17.5
90	0.3	958 (3.2)	43	447 (1.49)	23.5	342 (1.14)	18
100	0.333	1107 (3.7)	45	528 (1.76)	24	366 (1.22)	18.5

The table shows a range of daily diagnosed case(s) in the community, which our simulations use as school closure activation triggers. It relates the activation trigger to the proportion of population newly diagnosed per day, the cumulative number of diagnosed cases, and the consequent delay in intervention corresponding to each activation trigger, for three different simulated epidemics with R_0 _values of 1.5, 2.0 and 2.5.

For a number of daily diagnosed cases to count towards the intervention activation, it is assumed that the following sequence of events occurs:

i) The individual becomes infected with the pandemic strain.

ii) The individual experiences significant symptoms.

iii) They seek medical attention.

iv) The infection is identified as pandemic influenza strain.

v) The case should is reported to a public health monitoring scheme.

The diagnosis ratio (or ascertainment efficiency) is assumed such that 50% of the symptomatic cases should be diagnosed following the conditional probability that event e) occurs given that both a) and b) have occurred. A further assumption is that there are no false positive reports during pandemic influenza.

### Impact of individual school vs. simultaneous school closure interventions

The type of school closure (either *individual school closure *or *simultaneous school closure*) which gives the maximum reduction in attack rate also depends on R_0_, activation triggers and closure durations. The comparative effectiveness of both *individual school closure *and *simultaneous school closure *interventions are given in Table 2. Our simulation results suggest that *simultaneous school closure *is more effective if it is perfectly timed. However, the range of timings (activation triggers) that give the optimal reduction in attack rate can be narrow. *Individual school closure *is often capable of achieving almost the same reduction in attack rate, but over a wider range of activation triggers. For example, at R_0 _= 1.5, an ~8% (from 33% to 25%) reduction in attack rate would be achieved by 2 weeks *simultaneous school closure *when the number of daily diagnosed cases (activation trigger) reached 70, with almost the same effect being obtained within the range of 60 and 80 diagnosed cases per day (see Figure 1 for R_0 _= 1.5; blue lines with circle marker). However, the same ~8% reduction would be achieved by 2 weeks *individual school closure *when the number of daily diagnosed cases (activation trigger) reached 50, with almost the same effect being obtained within the wider range of 30 and 70 diagnosed cases per day (see Figure 1 for R_0 _= 1.5; blue lines with square marker). A detailed summary of the range of activation triggers for each school closure strategy, at which a maximum reduction in attack rate can be achieved, is given in Table 2.

**Table 2:** Optimal attack rate reductions and sensitivity to activation trigger for school closure strategies.

		School closure duration					
		
		2 weeks		4 weeks		8 weeks	
		
R_0_	Intervention	Attack rate	Activation trigger range	Attack rate	Activation trigger range	Attack rate	Activation trigger range
1.5	none	32.5		32.5		32.5	
	ISC	25.0	**30 - 70**	22.7	**20 - 50**	19.0	**10 - 30**
	SSC	24.7	60 - 80	22.4	40 - 60	18.3	20 - 30
	ISC + AV	17.3	**1 - 30**	16.0	**1-30**	14.0	**1-20**
	SSC + AV	17.0	30 - 40	15.9	20-30	13.8	20-30
2.0	none	49.9		49.9		49.9	
	ISC	45.0	**40 - 90**	43.0	**10 - 70**	41.0	1 - 10
	SSC	45.2	70 - 90	42.8	**30 - 90**	40.5	**10 - 30**
	ISC + AV	35.5	**1- 80**	33.6	**20 - 90**	31.6	1 - 10
	SSC + AV	35.3	70 - 90	33.4	50 - 90	31.5	**10 - 50**
2.5	none	58.8		58.8		58.8	
	ISC	55.8	**30 - 90**	54.8	1 - 20	54.5	**1 - 50**
	SSC	55.7	80 - 90	54.7	**10 - 90**	54.2	**1 - 50**
	ISC + AV	46.7	**30 - 90**	45.5	**1 - 90**	44.8	1 - 10
	SSC + AV	46.7	60 - 90	45.5	20 - 90	44.3	**1 - 30**

For each R_0 _value in, each school closure intervention (individual vs. simultaneous, with and without antiviral treatment and household prophylaxis), and each school closure duration (of 2, 4 or 8 weeks), this table gives: (a) the reduced attack rate attained by closing schools with the optimal time (i.e. using the optimal activation trigger), and (b) the range of activation triggers for which the attack rate reduction is almost the same as the optimal value. Attack rate is expressed in % of population. The range of school closure activation trigger as a reported number of daily diagnosed cases. The bold **activation trigger **indicates the wider range of a school closure strategy for which that strategy would be more effective i.e. the attack rate would be almost same. A diagnosis ratio of 50% is assumed.

### School closure and age-specific attack rates

**Figure 3:**
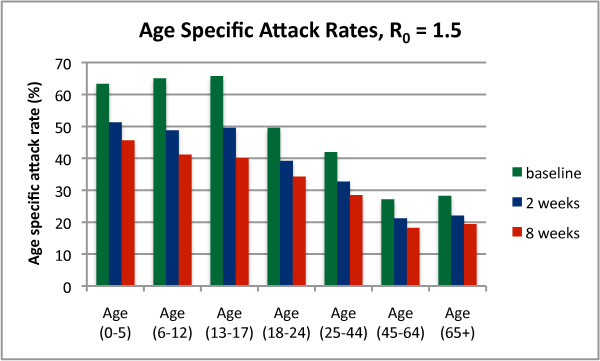
**Impact of school closure on age-specific attack rates**. Age specific attack rates (the proportion of each age group experiencing symptomatic infection) are shown for the baseline case (no interventions), 2 weeks school closure and 8 weeks school closure. The unmitigated epidemic has an R_0 _of 1.5. School closure is timed optimally according to policy recommendation in Table 4.

Although our simulated school closures applied directly to the 6-12 and 13-17 age groups, we found that reductions in attack rate were experienced by all age groups. Figure 3 shows age-specific attack rates for an epidemic with R_0 _of 1.5, for the baseline (no-intervention) case and for 2 and 8 weeks of school closure. Although proportional reductions in attack rates are largest in the school age groups (37% and 39% for the 6-12 and 13-17 groups respectively for 8 weeks school closure), the reductions in the other age groups are still considerable, ranging from 28% to 33% for 8 weeks school closure. As the school-age groups comprise only 20% of the population, the results indicate that 66% of cases avoided due to school closure occur outside the school age groups.

### Sensitivity analysis

Epidemiological data for pandemic influenza are often uncertain, sparse, limited and widely distributed. There are uncertainties in characteristics such as mortality, morbidity, disease transmissibility, contact behavior and behavioral changes among people during a pandemic and also in antiviral and vaccine efficacies, pre-existing immunities and well as other uncertainties. The simulation model upon which the current study is based has been subject to an analysis determining its sensitivity to alternative estimates for parameters relating to serial interval, age-specific attack rates and other assumptions (see [21], electronic supplementary material, Text S2). This analysis shows sensitivity of the un-mitigated attack rate and of the attack rate when mitigated by school closure. The current simulation model differs from the earlier model only in that it has a more refined model of viral shedding (see Methods, Transmission model).

**Figure 4:**
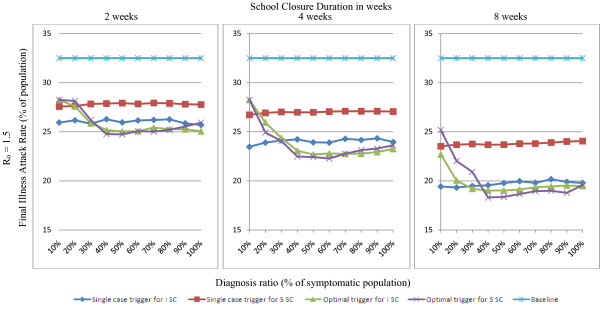
**Impact of diagnosis ratio on the effectiveness of school closure activation trigger for R_0 _= 1.5**. Outcomes of the activation trigger that would give the maximum reduction in attack rate (as *Optimal *trigger given in Table 3 assuming 50% *diagnosis ratio*) and the activation trigger at a single symptomatic case (*Single case *trigger) for *individual school closure *(*ISC*) and *simultaneous school closure *(*SSC*) strategies in relation to diagnosis ratio (% of symptomatic cases) are shown. The outcomes are reported in percentage of the simulated population size for the epidemics with R_0 _values of 1.5 regarding school closure durations of 2, 4 and 8 weeks.

In this study we tested the sensitivity of our results to the parameter known as *diagnosis ratio *(or case ascertainment efficiency) ranging from 10% to 100% of symptomatic cases being diagnosed during the epidemic period. The *diagnosis ratio *may be influenced by an efficient surveillance policy and has a significant impact on the effectiveness of interventions. The outcomes for different levels of diagnosis ratio for school closure strategies for an epidemic with a reproduction number R_0 _= 1.5 are given in Figure 4. In this figure, optimal activation triggers are used assuming a diagnosis ratio of 50%, but the true diagnosis ratio is allowed to vary.

The most notable outcome is that at least a 40% *diagnosis ratio *is required for the maximum reduction in attack rate (see Figure 4; green and purple line). A lower *diagnosis ratio *would result in an excessive delay of school closure activation. In contrast, *underestimating *the diagnosis ratio (and consequently introducing school closure too soon) has less serious consequences. The relationship between activation trigger and activation delay, assuming a *diagnosis ratio *of 50%, is given in Table 1.

## Discussion

### Key findings

**Figure 5:**
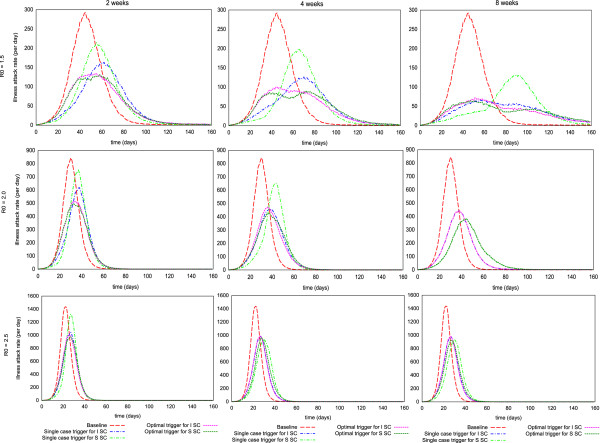
**Daily epidemic progression curves for different school closure activation triggers**. The daily incidence curves of the simulated epidemics (with R_0 _values of 1.5, 2.0 and 2.5) with the activation trigger that would be given the maximum reduction in attack rate (as *Optimal *trigger) and the activation trigger at a single symptomatic case (as *Single case *trigger) for *individual school closure *(*ISC*) and *simultaneous school closure *(*SSC*) strategies for 2, 4 and 8 weeks duration are shown. The red epidemic curves are for the baseline or un-mitigated epidemics for corresponding R_0_s. Blue and light green curves are for the *prompt *triggered ISC and SSC strategies. The other pink and dark green lines are for the *best *triggered ISC and SSC strategies.

This modelling work indicates that school closure strategies most effectively reduce both attack rate and peak daily incidence if they are initiated at the correct time - in this study, when a given number of cases have been diagnosed per day (see Table 3). A limited reduction in attack rate is obtained if closure activation occurs immediately (see Figure 1 and Figure 2). This phenomenon arises when school closure is triggered too early (at a single community-wide diagnosed case) and the school closure strategy is applied only once and for a fixed, limited closure duration, and is clearly illustrated in Figure 5 which shows epidemic curves for various school closure durations and activation triggers.

**Table 3:** Optimal school closure activation triggers

		Optimal triggers in the number of daily diagnosed cases per 30,000 population (% of population newly infected per day)		
		R_0_		
		
School closure strategy	Duration	1.5	2.0	2.5
**ISC**	**2 weeks**	50 (0.16)	80 (0.26)	80 (0.26)
	**4 weeks**	40 (0.13)	50 (0.16)	10 (0.03)
	**8 weeks**	20 (0.06)	1 (0.003)	1 (0.003)
**SSC**	**2 weeks**	70 (0.23)	80 (0.26)	80 (0.26)
	**4 weeks**	50 (0.16)	50 (0.16)	10 (0.03)
	**8 weeks**	30 (0.1)	10 (0.03)	1 (0.003)

For example, for an epidemic with an R_0 _of 1.5, with a 4 weeks school closure strategy (either *individual school closure *or *simultaneous school closure*) triggered by a single diagnosed case, the simulation results indicate that school closure slows the initial epidemic growth, but when school closure interventions are relaxed after 4 weeks the epidemic restarts (see Figure 5 for R_0 _= 1.5; blue and light green lines). Therefore, determining the optimal school closure trigger is crucial when the number of times schools close and their duration is limited. The optimal school closure activation triggers for a range of reproduction numbers, school closure types and duration scenarios are given in Table 3.

A greater number of activation triggers (for the reproduction numbers and durations considered) have the *individual school closure *strategy performing better (in terms of reduction in attack rate) than that involving *simultaneous school closure*. For the trigger scenarios where simultaneous closure is the better strategy, difficulty arises if the optimal case load trigger is missed, possibly due to under- or mis-diagnosis of cases. If this is the situation then individual school closure performs better, and hence is a safer strategy to adopt.

Given that at the beginning of an influenza pandemic we are unlikely to know how many asymptomatic or un-diagnosed cases exist, it may be difficult to determine when the optimal activation trigger has been reached. Since individual school closure does almost as well over a greater number of activation triggers, this suggests that it is a more reliable strategy to adopt in the face of uncertainty about the true degree of epidemic spread. Furthermore, individual school closure is more likely to be socially acceptable for mild pandemic strains, as parents will be aware of the local cases triggering the school closures that affect them.

The marginal increase in effectiveness of additional weeks of school closure decreases as the duration increases. For example, at R_0 _= 1.5, 2 weeks school closure gives an 8% reduction (4% per week) in attack rate. An additional 2 weeks school closure gives an additional 2% (1% per week) reduction. A further 4 weeks school closure (8 weeks total) gives an additional 4% (1% per week) reduction (see Figure 1 for R_0 _= 1.5; blue, orange and dark red lines with circle and square marker).

School closure and antiviral drug use for treatment and prophylaxis are complementary. The combination of antiviral strategies together with school closure *always *gives an increased reduction in attack rate compared to either in isolation, across different transmissibility characteristics, school closure activation triggers (except for significantly delayed school closure at R_0 _= 1.5), school closure types and school closure durations (see Figure 1 for R_0 _= 1.5).

### Modelling features and relationship to other studies

We have developed an individual-based simulation model of Albany (a small city of ~30,000 populations in Western Australia) utilizing Australian Bureau of Statistics Census data [31] to simulate virtual epidemics with a range of characteristics and to systematically evaluate school closure strategies. Related models ranged in scale from the whole world [32], through large [17,18] and smaller [16,33] countries, to actual [21] and synthetic [19,10] small communities. Our model differs from the differential equations-based deterministic and compartmental models [34-37] which consider homogeneous mixing patterns within and between subpopulations; for a survey on mathematical modelling of influenza pandemics see [38]. Our model encompasses significant complexity including spatial contact structure; age specific susceptibility; mixing groups and community wide random contacts [21,23]. For the purposes of this study the model has been augmented with sophisticated models of school closure and antiviral prophylaxis and treatment.

Empirical evidence and previous modelling studies indicate that school closure can reduce the magnitude of an influenza epidemic; for a review see [9]. The effectiveness of school closure clearly depends on operational issues such as *what type *of school closure is adopted (*simultaneous school closure *or *individual school closure*), *when *school closure should be triggered, and *how long *schools should be closed.

Previous modelling studies have made particular assumptions, plausible or idealized, about these issues, and found school closure to be effective to a lesser or greater extent. In Ferguson *et al. *[17] 3 weeks of *individual school closure *strategy was chosen for multiple times and the strategy was activated after a single community case was detected. In Germann *et al. *[18], Glass *et al. *[19] and Davey *et al. *[20,39], *simultaneous school closure *strategy was assumed for whole pandemic period with an activation trigger following single or several community case(s) detection (in Germann *et al. *triggered at 1 community case, in Glass *et al. *at 10 community case and in Davey *et al. *a sensitivity for 10, 30 and 100 community cases was shown). In Milne *et al. *[21] a comparison of school closure interventions evaluated using different individual-based models was studied. In that study the authors emphasized the benefit of long duration school closure in containing an influenza pandemic. However long term school closure has an adverse economic impact; Sadique *et al. *[24] estimated a cost in the range 0.2% - 1.0% of GDP in the UK following a 12 weeks of school closure. The effectiveness of school closure evaluated in these modelling studies depended largely on the underlying assumptions made about their models.

In this study we have explicitly considered three key school closure operational issues which were identified in [9] as being significant. These are *when *school closure should be initiated (activation trigger), *how long *schools should be closed (duration) and *what type *of school closure should be adopted and determined their impact on influenza pandemics: no previous modelling study has systematically evaluated such school closure operational issues. We believe that the detailed evaluation of such strategies will contribute to further development of school closure policy guidelines for future influenza pandemics.

Antiviral drug strategies also play an important role in controlling disease spread at the early stages of an outbreak. In addition to school closure (in isolation), we simulated the application of antiviral drug treatment and prophylaxis to household members of an infected case layered with school closure. A detailed evaluation of the effectiveness of antiviral drug strategies applied to a pandemic similar to the A/H1N1 2009 influenza pandemic has been investigated in [26].

There are some limitations of the model used in this study. As the model is based on a population in a developed country the outcomes may not be applicable to populations in a developing country, where populations may be less mobile and have higher population densities. We have focused on the reduction in the number of daily symptomatic cases and the cumulative illness attack rate as they are used for determining intervention effectiveness rather than focusing on influenza-related adverse events such as hospitalizations and deaths. We also do not take account of possible antiviral drug resistance [40,41] that may arise due to the implementation of antiviral drug strategies, as our main goal is to suggest refinements to policy guidelines for school closure.

### Public health policy implications

We have evaluated a range of school closure activation triggers (as a function of the reported number of daily diagnosed cases in a community), school closure durations, and types of school closure interventions for the potential control of a future influenza pandemic. The results may be used to inform public health authorities as they revise guidelines for school closure. Although short periods of school closure strategies were adopted in different countries as an attempt at controlling spread the A/H1N12009 influenza pandemic [11,14,15,42], they met with limited success; for a highly pathogenic influenza pandemic longer periods of school closure would need to be adopted (see Table 4) as school closure policy recommendations. Our results imply that closure periods longer than 2 weeks may be effective in giving significant reduction in attack rate (see Figure 1; for R_0 _= 1.5).

**Table 4:** School closure policy recommendations

		Pandemic transmissibility		
		
Pandemic severity	School closure duration	low (R_0 _= 1.5)	medium (R_0 _= 2.0)	high (R_0 _= 2.5)
**mild**	**2 weeks**	Schools should be closed individually when cases are identified in each school; this policy should delayed until the first day on which **13 new cases per 10,000 population **are diagnosed (assuming 50% diagnosis of symptomatic individuals).		

**moderate**	**4 weeks**	Schools should be closed individually when cases are identified in each school; this policy should be instituted as soon as possible once the pandemic has reached the community. Antivirals should be dispensed to slow the spread.		

**severe**	**8 weeks**	Schools should be closed individually when cases are identified in each school; this policy should delayed until the first day on which **6 new cases per 10,000 population**are diagnosed (assuming 50% diagnosis rate). However, if the activation trigger is missed *all *schools should close as soon as possible. Antivirals should be dispensed together with school closure and home isolation.	All schools should close simultaneously as soon as possible once the pandemic reaches the community. Antivirals and other non-pharmaceutical interventions should also be applied.	All schools should close simultaneously as soon as possible once the pandemic reaches the community Antivirals should be dispensed in larger extent to slow down disease spread. Other social distancing based interventions should be rigorously applied.

We assume that a key factor which will determine the duration of school closure will be the perceived severity of the pandemic (i.e. morbidity and mortality given as case hospitalization and case fatality ratios). For a 'mild' pandemic similar to the A/H1N1 2009 pandemic and similar in severity to seasonal influenza [43], it is unlikely that school closure would be tolerated for periods longer than two weeks. In contrast, during a pandemic with severity similar to the 1918 pandemic (case fatality ratio estimated as > 2.5%) [1], the public might accept (or even demand) school closure for several months.

Recommendations for school closure interventions should reflect that in the case of a *severe *pandemic with high transmissibility (R_0 _>= 2.0), where schools would be closed for at least 8 weeks, all schools should be closed at the same time, as early as possible once the epidemic reaches the local community. In the case of a *moderately severe *pandemic (case fatality ratio < 1.0%) where schools would be closed for at least 4 weeks, schools should close individually once cases are detected in each school and this policy should be instituted as soon as possible. In the case of a *mild *pandemic similar to the A/H1N1 2009, where at most 2 weeks of school closure would be adopted, schools should close individually, but the policy should be delayed until the first day on which 0.13% of the population becomes newly infected (on that day), assuming 50% of symptomatic cases would be diagnosed (see Table 4 for a set of possible school closure policy recommendations).

The additional use of antiviral treatment and prophylaxis will give a greater reduction in attack rate than school closure in isolation for influenza epidemics with R_0 _>= 2.0, (see Figure 1 for R_0 _>= 2.0).

For epidemics with R_0 _<= 1.5 where antivirals are used, there is no advantage having periods longer than 2 weeks (eg. 4 or 8 weeks) of school closure *if school closure is delayed for 40 days or more *(see Figure 1 for R_0 _= 1.5). In all other scenarios, the combination of antivirals and school closure is substantially more effective than either strategy alone. For highly transmissible epidemics (R_0 _= 2.5), fixed period school closure alone will be ineffective (i.e. have a limited ~4% reduction in the attack rate) (see Figure 1 for R_0 _= 2.5); therefore, additional rigorous social distancing based interventions would need to be applied [21,23]. If the virus strain is matched to stockpiled vaccines then vaccination strategies suggested in [25] should be applied at an early stage.

Selecting a school closure activation trigger for short closure periods using a reported number of diagnosed cases per day (rather than a fixed period of time, say 2 weeks) means that the timing adapts to faster developing epidemics (those with higher reproduction numbers). For example, the 40 daily diagnosed cases per 30,000 populations occur at 33, 20 and 15 days for R_0 _= 1.5, 2.0 and 2.5 respectively (see Table 1). These results show that the optimal use of school closure depends both on pandemic severity (which will determine the feasible duration of school closure) and its transmissibility characteristics.

Our approach to modelling a generic influenza pandemic was to base epidemic characteristic on those of seasonal influenza strains (for which data is available), but to increase the overall transmissibility (by increasing the basic transmission probability) in order to represent higher infectivity of immunologically novel strains. We thus assumed that the age specificity of infection followed a pattern typical of seasonal influenza. With the advent of the 2009/2010 pandemic, data from an actual pandemic influenza strain has become available, albeit a strain that has turned out to be less pathogenic and immunologically novel than first thought. Our R_0 _= 1.5 simulations align with estimates for the 2009 pandemic [44,42-47], as does our derived serial interval of 2.49 days. The A/H1N1 2009 pandemic exhibited a somewhat different pattern of age-specific infectivity than our seasonal influenza baseline, with higher attack rates in children and young adults but lower attack rates in older adults. We conducted an alternate set of simulations based on the A/H1N1 2009 age-specific attack rate pattern and R_0 _(results not reported here)_. _We found that the alternative assumptions lead to a lower overall attack rate (compared to a "seasonal" epidemic with the same R_0_), but that the proportional effectiveness of school closure and optimal activation triggers were essentially identical.

## Conclusions

Our simulation results give guidance as to public health policy decisions in the refinement of school closure strategies to be used in a future influenza pandemic. We have systematically evaluated school closure operational issues to determine when schools should be closed and re-opened to achieve the maximum reduction in influenza spread. We found that the optimal timing of school closure depends both on the duration of school closure (which we assume will depend on the severity of the influenza strain, with strains that are more severe in terms of serious infection outcomes making longer periods of school closure acceptable) and on the transmissibility of the influenza strain (which influences the rate of growth and spread of the epidemic). Accurate early estimates of epidemic characteristics such as the basic reproduction number and disease severity are thus necessary to achieve the maximum case reduction from school closure. We found that a policy of allowing schools to close individually was much less sensitive to the precise timing of the intervention than a policy of simultaneous community-wide school closure, a valuable observation given the difficulty in determining the true degree of epidemic spread in the early stages of an outbreak.

## Competing interests

The authors declare that they have no competing interests.

## Authors' contributions

NH, JK and GM were responsible for the conception and design of the simulation experiments. NH and JK were responsible for software development. NH conducted simulation experiments. All authors were involved in the analysis of simulation results and writing the manuscript.

## Pre-publication history

The pre-publication history for this paper can be accessed here:

http://www.biomedcentral.com/1471-2334/10/221/prepub
